# The Effect of Antiretroviral Treatment on Health Care Utilization in Rural South Africa: A Population-Based Cohort Study

**DOI:** 10.1371/journal.pone.0158015

**Published:** 2016-07-06

**Authors:** Jan A. C. Hontelez, Frank C. Tanser, Kevindra K. Naidu, Deenan Pillay, Till Bärnighausen

**Affiliations:** 1 Wellcome Trust Africa Centre for Population Health, University of KwaZulu-Natal, Mtubatuba, South Africa; 2 Department of Global Health and Population, Harvard T.H. Chan School of Public Health, Boston, United States of America; 3 Department of Public Health, Erasmus MC, University Medical Center Rotterdam, Rotterdam, Netherlands; 4 School of Nursing and Public Health, University of KwaZulu-Natal, Durban, South Africa; 5 Division of Infection and Immunity, University College London, London, United Kingdom; University of Washington, UNITED STATES

## Abstract

**Background:**

The effect of the rapid scale-up of vertical antiretroviral treatment (ART) programs for HIV in sub-Saharan Africa on the overall health system is under intense debate. Some have argued that these programs have reduced access for people suffering from diseases unrelated to HIV because ART programs have drained human and physical resources from other parts of the health system; others have claimed that the investments through ART programs have strengthened the general health system and the population health impacts of ART have freed up health care capacity for the treatment of diseases that are not related to HIV. To establish the population-level impact of ART programs on health care utilization in the public-sector health system, we compared trends in health care utilization among HIV-infected people receiving and not receiving ART with HIV-uninfected people during a period of rapid ART scale-up.

**Methods and Findings:**

We used data from the Wellcome Trust Africa Centre for Population Health, which annually elicited information on health care utilization from all surveillance participants over the period 2009–2012 (N = 32,319). We determined trends in hospitalization, and public-sector and private-sector primary health care (PHC) clinic visits for HIV-infected and -uninfected people over a time period of rapid ART scale-up (2009–2012) in this community. We regressed health care utilization on HIV status and ART status in different calendar years, controlling for sex, age, and area of residence. The proportion of people who reported to have visited a public-sector primary health care (PHC) clinic in the last 6 months increased significantly over the period 2009–2012, for both HIV-infected people (from 59% to 67%; p<0.001), and HIV-uninfected people (from 41% to 47%; p<0.001). In contrast, the proportion of HIV-infected people visiting a private-sector PHC clinic declined from 22% to 12% (p<0.001) and hospitalization rates declined from 128 to 82 per 1000 PY (p<0.001). For HIV-uninfected people, the proportion visiting a private-sector PHC clinic declined from 16% to 9%, and hospitalization rates declined from 78 to 44 per 1000 PY (p<0.001). After controlling for potential confounding factors, all trends remained of similar magnitude and significance.

**Conclusions:**

Our results indicate that the ART scale-up in this high HIV prevalence community has shifted health care utilization from hospitals and private-sector primary care to public-sector primary care. Remarkably, this shift is observed for both HIV-infected and -uninfected populations, supporting and extending hypotheses of ‘therapeutic citizenship’ whereby HIV-infected patients receiving ART facilitate primary care access for family and community members. One explanation of our findings is that ART has improved the capacity or quality of primary care in this community and, as a consequence, increasingly met overall health care needs at the primary care level rather than at the secondary level. Future research needs to confirm this causal interpretation of our findings using qualitative work to understand causal mechanisms or quasi-experimental quantitative studies to increase the strength of causal inference.

## Introduction

The rapid scale-up of antiretroviral treatment (ART) for HIV in sub-Saharan Africa (SSA) over the past decade is an unprecedented achievement in the global fight against HIV and one of the largest public health achievements in recent history. Due to large investments from sources such as the Global Fund to Fight AIDS, Tuberculosis, and Malaria; the Presidents Emergency Plan for AIDS Relief (PEPFAR); and bilateral donors, the number of HIV-infected people receiving life-saving treatment in SSA increased from just 300,000 in 2004, to 7.5 million in 2012 [1]. As a consequence, HIV related morbidity and mortality has fallen dramatically on the continent [1]. South Africa is home to the largest HIV epidemic worldwide, with about 6 million people living with HIV [1]. Here, the rapid scale-up ART since 2004 resulted in a total of about 2.5 million people on HIV treatment in 2014, and has substantially improved the health of HIV-infected people and increased general life expectancy [2–4].

Despite the impressive population health gains due to large-scale HIV treatment and care [2], the broader impact on the health system in general is still under intense debate [5–8]. HIV treatment is mostly delivered through ‘vertical’, i.e., disease-specific, treatment and care programs [9]. These programs provide ART separately from the general ‘horizontal’ health care system, which is charged with addressing the other health burdens affecting the South African population [9]. The large scale investments in HIV treatment have thus been criticized for displacing funding, human resources, and infrastructure for other health care, and further weakening the already vulnerable health systems by directing very large proportions of overall resources to the treatment of a single, increasingly chronic, disease [10–12]. In contrast, others have argued that HIV treatment programs are beneficial to the general health system, as large parts of financial investments contribute directly to supporting essential components of health systems that can be utilized for a wide range of health care activities, such as human resources training, building infrastructure, and improving monitoring and evaluation capacity [13,14]. The South African health system is currently overburdened, and inequalities in access are high, as recognized in the National Health Insurance plan [15]. Health care access is particularly low for poor, black South Africans in rural areas [16], while wealthier people have access to the comparatively expensive private health care system in the country [17,18].

There are several ways in which the ART scale-up could affect the health system. As successful ART reduces both morbidity and mortality in HIV-infected individuals [19], ART may reduce utilization of secondary levels of care (e.g. hospitalization) in this population, freeing up scarce higher level health care resources for other use. In addition to this supply-side effect benefiting the entire population, it is also plausible that ART may have had positive demand-side effects on general health care utilization. The latter involves mechanisms similar to those described for the process of ‘therapeutic citizenship’, in which HIV-infected people appropriate ART as a set of rights and responsibilities, and as a result act as advocates of ART [20,21]. Similarly, people on ART may also act as facilitators for HIV-uninfected family and community members to visit the public-sector PHC clinics through empowerment; through visual effects of improved health of people on ART improving the perception of quality of the public-sector health care system; through direct contacts established via HIV-infected family members on ART; and through improved physical access.

We investigate the effects of ART treatment and care programs on the health system by comparing trends of health care utilization during a time of rapid ART scale-up in both HIV-infected and -uninfected people. The population-based longitudinal cohort run by the Wellcome Trust-funded Africa Centre for Population Health in rural KwaZulu-Natal (Africa Centre), South Africa, provided us with the rare opportunity to analyze population-based health care utilization trends in a time of rapid ART scale-up both overall and by HIV status. In this cohort, the total population living in a geographically circumscribed area in the Hlabisa sub-district of South Africa (more than 100,000 individuals living in about 11,000 households) are under continuous longitudinal surveillance regarding a range of health care and social intervention exposures as well as health, economic, social and behavioral outcomes [22]. The HIV treatment and care program in the sub-district where the Africa Centre’s population-based cohort is located is run by the Department of Health (DoH) and delivers ART through 17 nurse-led public-sector stand-alone primary health care (PHC) clinics. The program, which started in 2004, was never hospital-based and was from the start decentralized to PHC clinics. The number of clinics included in the program increased over the period 2004–2007. Initially, 2 PHCs started offering ART in early 2004; by early 2005, the number of PHC clinics offering ART had increased to 14; and by early 2008, all 17 PHC clinics in the Hlabisa sub-district had operational HIV treatment and care programs [23]. During the observation period of this study (2009–2012), these clinics were vertically organized—with their own staff, patient records, supply chain, management, and monitoring and evaluation system—but they were located in immediate proximity to the ‘horizontal’ health care system on the premises of the 17 public-sector PHC clinics in the sub-district. The HIV treatment and care program has rapidly increased ART coverage in the Africa Centre surveillance community [24], resulting in large life expectancy gains [3]. In the pre-2009 period, coverage increased from 0.0% in 2004 to 14.3% in 2008 [25]. The local district hospital (Hlabisa hospital), is a 300-bed public sector hospital. ART is available both through public- and private-sector clinics. However, private sector ART utilization in this community is overall very limited: Most HIV patients lack health insurance and thus have to pay substantial user fees when utilizing ART through private clinics. The vast majority of HIV patients thus chooses to access ART through public-sector clinics, where the treatment is free of charge. There are no private-sector clinics in the Africa Centre surveillance area itself. The private-sector clinics that are closest to the surveillance area are located in the market town of Mtubatuba. These private clinics are operated and owned by single general practitioners, internists or pediatricians, providing a broad range of general health care services [23].

We used data from the Africa Centre to determine trends in public-sector and private-sector PHC clinic visits and hospitalization rates in the general population during a time of rapid ART scale-up.

## Methods

### Setting and data

We used data from the Africa Centre population-based cohort [22]. The cohort area covers about 438 km^2^ [22,26] and over 100,000 largely Zulu speaking people, of which the majority live in scattered homesteads throughout the area [26]. The area is characterized by a high HIV prevalence and high ART uptake. In 2011, HIV prevalence in adults was nearly 30% [25], and about 18,000 people were initiated on ART as of end-2012. Over the study observation period, the Hlabisa HIV Treatment and Care Programme was run by DoH with support from the Africa Centre through large grants from the US Presidential Emergency Fund for AIDS Relief (PEPFAR). The program covers the entire Hlabisa sub-district with a population of about 228,000 people [23], which is located in uMkhanyakude district, the poorest among the 52 districts in the nation. The Africa Centre population-based cohort covers about half of the geographical area and population in the sub-district, which generates the opportunity for individual-level linkage of data from the population-based cohort and the clinical cohort data.

The Africa Centre population-based cohort includes individual and household surveys. The household surveys consist of a set of questionnaires administered to the head or key informant of the household. They contain questions on events that change the household structure, such as birth, migration, marriage, or death, as well as a wide range of economic and social exposures and outcomes. These surveys were conducted every 6 months during the study period. In addition, individual health surveys are conducted once a year in the entire adult population living in the cohort area. The individual surveys include HIV sero-surveillance (since 2003/2004) and questions on a range of characteristics and behaviors. Household and individual surveys are linked longitudinally to each other and over time through unique individual and household identification numbers. The different surveys are described in detail elsewhere (http://www.africacentre.ac.za/Default.aspx?tabid=69) [22]. Since 2009, the individual surveys have included questions on health care utilization: (1) the number of hospital visits in the last 12 months, (2) whether an individual person visited a public-sector PHC clinic in the past 6 months, and (3) whether an individual visited a private-sector PHC clinic in the last 6 months.

We identified the ART status of all individuals in the Africa Centre population-based cohort through linkage of the cohort data to the patient data from Hlabisa HIV Treatment and Care Programme, using the South African national identification (ID) number, and other individual identifiers, such as birth date, first name and last name.

The program offers HIV treatment and care; routinely collected data in all patients and at each time point includes dates of all clinic visits within the program, CD4 cell count, viral load, and HIV drug regimen. All patients are required to return to the clinic every month and are seen by a professional nurse and an HIV counselor. CD4 counts and viral loads are routinely collected every 6 months. When patients have not reported back to the clinic for a period of 3 months or longer, they are recorded as being lost-to-follow up in the program, but data on these patients continues to be available through the Africa Centre population-based cohort data collection. The HIV treatment and care program is described in more detail elsewhere [23,27].

The main exposure variable in our analysis was the year of observation (2009 to 2012), as calendar time in this setting is a good proxy for the scale of the ART roll-out [2,28,29], and there were no major changes in the health system other than the ART program during the study period. In addition, we controlled for the following variables in multivariable regression: HIV status (uninfected/infected), ART status (not on ART/On ART < 6 months/On ART ≥ 6 months/unknown), observation year, age, age square, and area of residence (within the demographic surveillance area (DSA): rural/peri-urban/urban, and outside the DSA).

### Ethics statement

Informed written consent was obtained from all adult eligible persons aged 15 year or older for participation in the individual health surveillance and to provide a small blood sample for HIV analysis for research purposes. As permitted by the regulatory framework governing research in South Africa at the time of the study, we obtained written informed consent from adolescents aged 15–17 years themselves. Similar to other HIV surveys and surveillance, such as the DHS, the individual health surveillance did not reveal HIV results to participants, but instead provides information on location and opening hours of the public-sector HIV counselling and testing facilities, where rapid HIV tests are offered free of charge.

The obtained information on ART status by linking study participants with the local HIV treatment and care programme database, which is housed at the Africa Centre, and which information can be linked to that of the individual health surveillance at an individual level using a range of variables including surname, first names, date of birth, sex, South African I.D. number, closest clinic, mother’s name and date of death. After linkage, all individual level data were de-identified to prevent analysts working with the data from identifying any of the individuals. Participant names and South African identification numbers are replaced with an anonymous surveillance system number, which analysts cannot link back to the individual participants.

Ethical approval for the individual health surveillance (reference BF233/09) and for the linkage between the individual health surveillance and HIV treatment and care programme databases (reference E134/06) was obtained from the Biomedical Research Ethics Committee (BREC) of the University of KwaZulu-Natal, and renewed on an annual basis. The BREC was aware that some of the surveillance participants were minors and approved the age range and consent procedure of participation. All data were anonymized before they were accessed by the researchers.

### Statistical analyses

First, we determined crude trends in self-reported health care utilization rates over the four-year period 2009–2012 in the adult population (aged 15 years and above). We performed overall trend analysis of public-sector PHC clinic visit rates, private-sector PHC clinic visit rates, and hospitalization rates, stratified by HIV status (uninfected, infected, unknown). We standardized self-reported health care utilization rates using direct age and sex standardization in order to control for changes in the age composition of the participants. We used a Pearson chi-squared test for trends to determine significance.

Next, we regressed the clinic visit and hospitalization rates on HIV status and ART status. We used multivariable mixed-effects models, with individual random effects to control for dependence of repeated observations in the same individual in addition to calendar time fixed effects. We used logistic regressions for the binary outcomes indicating whether an individual had in the past 6 months visited a public-sector PHC clinic and whether he or she had visited a private-sector PHC clinic; we used Poisson regression for the count data on individuals’ hospitalization rates. We added control variables in nested regression models. We performed likelihood tests to determine whether the addition of control variables improve the statistical fit of the regression equations.

We present health care utilization rates by HIV status and observation year. Utilization in HIV-uninfected people in 2009 was chosen as reference category in order to facilitate comparisons of utilization across HIV status, ART status, and calendar year categories. We chose this approach to categorizing the different exposure categories, because they allow comparison of clinic visits and hospitalization rates across the different HIV status and ART status categories, rather than merely within each of these categories. All analyses were done with Stata 13.0.

## Results

Table 1 gives an overview of the baseline characteristics of the study participants. There were a total of 32,319 unique individuals who completed the general health survey at least once in the period 2009–2012. Of these, 16,131 (49.6%) completed more than one survey, and 3,996 (12.3%) participated each year. The majority of participants were female (62.7%), and women were more likely to participate in multiple rounds. The proportion of participating men increased over time, from 29.0% in 2009 to 34.8% in 2012. ART coverage of all HIV-infected people in the area increased significantly, from 28% in 2009 to 47% in 2012 (p<0.001; Chi^2^ test for trend). The overall hospitalization rate was 63.8 per 1000 person-years (PYs), and there was a strong decline in hospitalization rates over the period 2009–2012, from 87.3 to 62.6, 61.4, and 45.9 per 1000 PY respectively (p<0.001; Chi^2^ test for trend).

**Table 1:** Baseline characteristics of the participants.

	2009 (n = 13,500)	2010 (n = 14,472)	2011 (n = 15,136)	2012 (n = 14,713)	p-value
Sex (%)					
Women	9,588 (71.0%)	9,618 (66.5%)	9,969 (65.9%)	9,595 (65.2%)	<0.001
Men	3,912 (29.0%)	4,854 (33.5%)	5,164 (34.1%)	5,118 (34.8%)	
Median age [IQR]	30 [21; 49]	30 [20; 50]	31 [20; 51]	30 [19; 51]	0.8
HIV and ART status					
HIV-	7,760 (56.8%)	9,171 (63.4%)	9,014 (59.6%)	7,422 (50.5%)	<0.001
HIV+, not on ART	1,706 (12.6%)	2,051 (14.2%)	1,934 (12.8%)	1,600 (10.9%)	
HIV+, on ART	635 (4.7%)	854 (5.9%)	1,068 (7.1%)	1,336 (9.1%)	
HIV status unknown	3,489 (25.8%)	2,396 (16.6%)	3,120 (20.6%)	4,355 (29.6%)	
*ART coverage (95% CI)*	27.2% (25.4%; 29.0%)	29.4% (27.7%; 31.1%)	35.6% (33.9%; 37.3%)	45.5% (43.7%; 47.3%)	<0.001
Clinic visit in last 6 m (%)					
Yes	5,819 (43.6%)	6,851 (47.3%)	7,759 (51.3%)	7,300 (49.6%)	<0.001
No	6,903 (51.1%)	7,586 (52.4%)	7,341 (48.5%)	7,379 (50.2%)	
Missing	706 (5.2%)	35 (0.2%)	36 (0.2%)	34 (0.2%)	
Hospitalization rate per 1000 PY (95% CI)	87.3 (78.4; 96.1)	62.6 (57.8; 67.4)	61.4 (56.8; 65.9)	45.9 (41.7; 50.2)	<0.001
Area of residence					
Rural	7,996 (60.0%)	8,485 (58.9%)	8,882 (58.9%)	8,572 (58.3%)	<0.001
Peri-urban	3,392 (25.5%)	4,409 (30.6%)	4,580 (30.4%)	5,126 (35.9%)	
Urban	595 (4.5%)	704 (4.9%)	773 (5.1%)	933 (6.3%)	
Outside DSA	1,340 (10.1%)	808 (5.6%)	855 (5.7%)	74 (0.5%)	

**Fig 1:**
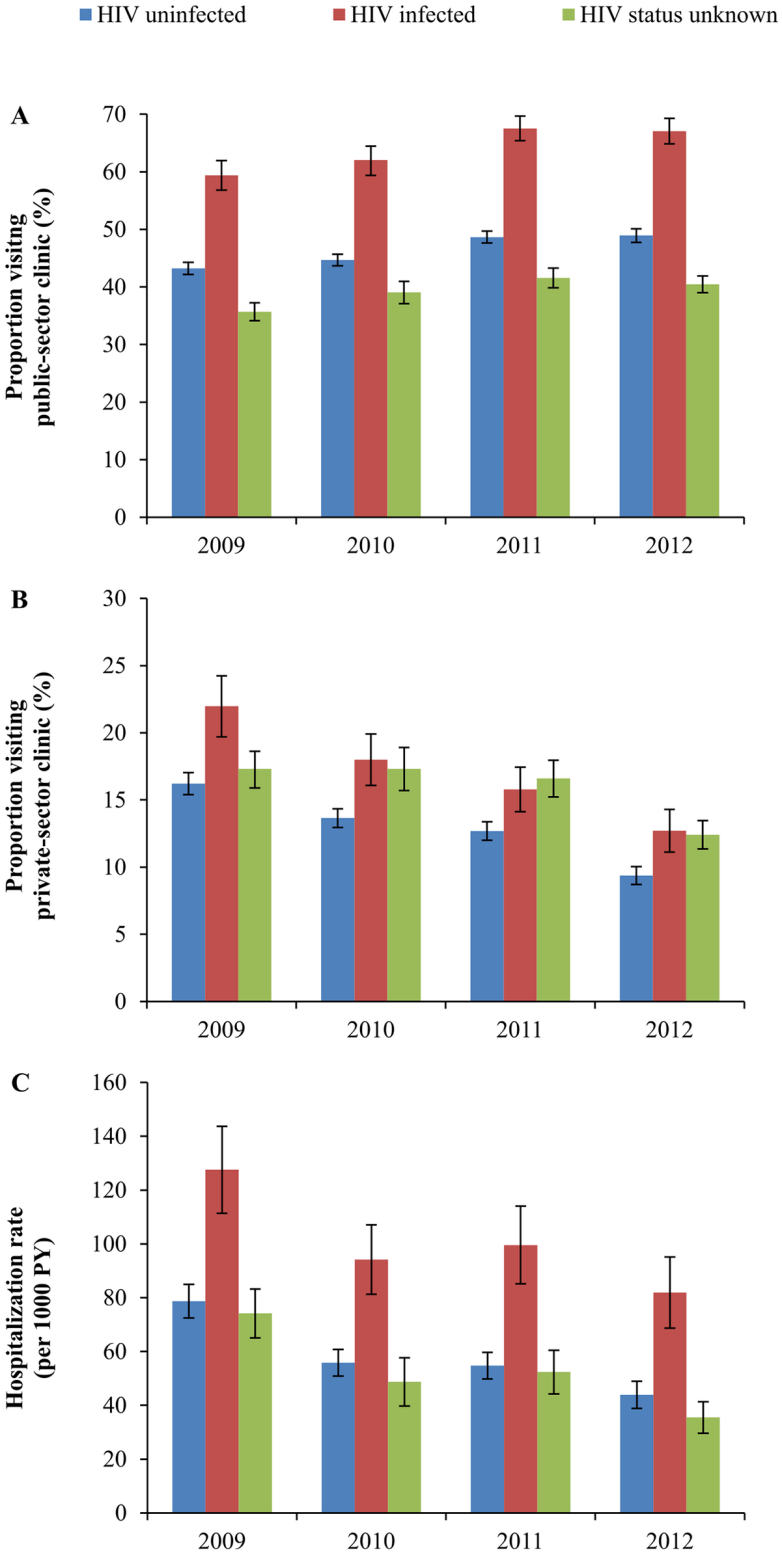
Trend in age-standardized self-reported health care utilization by HIV status over the years 2009 to 2012 in rural KwaZulu-Natal, South Africa. **A.** Proportion of people reporting to have visited a public-sector PHC clinic in the last 6 months. **B.** Proportion of people reporting to have visited a private-sector PHC clinic in the last 6 months. **C.** Self-reported hospitalization rates over the last 12 months. The public-sector ART program started in 2004. Pre-2009 trend in ART coverage was as follows: 0.0% in 2004; 1.0% in 2005; 3.8% in 2006; 8.3% in 2007; 14.3% in 2008 [26]. Coverage in the study period was as follows: 27.2% in 2009; 29.4% in 2010; 35.6% in 2011; and 45.5% in 2012 (Table 1).

Trends over time in the age-standardized proportions of people visiting a public-sector and private-sector PHC clinic, and age-standardized hospitalization rates by HIV status are shown in Fig 1. There was a significant increase in the proportion of people who visited a public-sector PHC clinic over the past 6 months for both HIV-infected and -uninfected people over the period 2009–2012 (Fig 1A): from 59% to 67% (p<0.001; chi^2^ test for trend) for HIV-infected; from 41% to 47% (p<0.001; chi^2^ test for trend) for HIV-uninfected people; and from 35% to 40% (p<0.001; chi^2^ test for trend) for people with unknown HIV status.

Coinciding with increasing rates of public-sector PHC clinic visits, there were clear and significant downward trends in the age-standardized proportions of the population visiting a private-sector PHC clinic (Fig 1B) and in hospitalization rates (Fig 1C). The proportion of HIV-infected people visiting a private-sector PHC clinic declined from 22% to 13% (p<0.001), and for HIV-uninfected private-sector PHC clinic visits declined from 16% to 9% (p<0.001), while for people with unknown HIV status this was from 17% to 12% (p<0.001). Hospitalization rates significantly declined for both HIV-uninfected, HIV-infected people, and people with unknown HIV status. For HIV-infected people, rates declined by about a third, from 82 per 1000 PY in 2012 compared to 127 per 1000 PY in 2009 (p<0.001). In addition, hospitalization rates nearly halved for HIV-uninfected people, from 78 to 44 per 1000 PY (p<0.001). Hospitalization rates declined from 74 per 1000 PY in 2009 to 35 per 1000 PY in 2012 for people with unknown HIV status.

The results of the multivariable regressions on health care utilization are shown in Table 2, and the nested models are given in S1 Table. All trends over time remained similar when we controlled for age, sex, and area of residence. Women were substantially and significantly more likely to utilize public-sector PHC, private-sector PHC, and hospital care. Health care utilization also increased significantly with age, but the rate of increase declined with increasing age. People living in peri-urban or urban areas were slightly less likely to have visited a public-sector PHC clinic compared to people living in rural areas (adjusted odds ratio (AOR) = 0.94; p = 0.008 and 0.81; p<0.001 respectively). In contrast, compared to people living in rural areas, people living in peri-urban and urban areas were more likely to have visited a private-sector PHC clinic (AOR = 1.18; p<0.001 and 2.06; p<0.001 respectively), and were more likely to be hospitalized (adjusted incidence rate ratio (AIRR) = 1.15; p = 0.008 and 1.53; p<0.001 respectively). Public-sector PHC clinic visit rates for people on ART for 6 months or longer increased slightly from 2009 to 2011, before decreasing in 2012; while private-sector PHC clinic visit rates and hospitalization rates declined substantially for the same population.

**Table 2:** Multivariable regressions of health care utilization on HIV status, ART status, duration on ART, and calendar year.

Parameter	Public-sector PHC clinic visits		Private-sector PHC clinic visits		Hospitalization rate	
	AOR (95% CI)	p-value	AOR (95% CI)	p-value	AIRR (95% CI)	p-value
HIV -						
2009	1	-	1	-	1	-
2010	1.06 (0.99; 1.12)	0.085	0.80 (0.74; 0.87)	<0.001	0.70 (0.59; 0.83)	<0.001
2011	1.28 (1.20; 1.36)	<0.001	0.72 (0.66; 0.78)	<0.001	0.68 (0.58; 0.81)	<0.001
2012	1.24 (1.16; 1.32)	<0.001	0.52 (0.47; 0.57)	<0.001	0.52 (0.43; 0.64)	<0.001
HIV +, no ART						
2009	1.30 (1.16; 1.46)	<0.001	1.24 (1.08; 1.42)	0.002	1.30 (0.99; 1.72)	0.062
2010	1.49 (1.34; 1.65)	<0.001	1.01 (0.88; 1.15)	0.918	1.01 (0.81; 1.27)	0.918
2011	1.71 (1.53; 1.91)	<0.001	0.90 (0.78; 1.03)	0.121	0.85 (0.69; 1.06)	0.152
2012	1.69 (1.50; 1.90)	<0.001	0.66 (0.56; 0.78)	<0.001	0.76 (0.58; 0.99)	0.041
On ART ≤6m						
2009	4.96 (2.94; 8.37)	<0.001	2.15 (1.41; 3.30)	<0.001	1.80 (1.35; 4.85)	0.041
2010	6.55 (3.80; 11.30)	<0.001	1.48 (0.94; 2.32)	0.092	2.56 (1.35; 4.85)	0.004
2011	11.53 (5.97; 22.27)	<0.001	1.85 (1.22; 2.82)	0.004	2.18 (1.37; 3.46)	0.001
2012	5.30 (3.24; 8.66)	<0.001	0.93 (0.58; 1.49)	0.766	1.78 (1.07; 2.95)	0.025
On ART > 6m						
2009	3.86 (3.07; 4.87)	<0.001	1.31 (1.06; 1.62)	0.013	2.67 (1.80; 3.94)	<0.001
2010	4.81 (3.91; 5.92)	<0.001	0.89 (0.73; 1.09)	0.257	1.27 (0.94; 1.71)	0.120
2011	7.26 (5.90; 8.94)	<0.001	0.80 (0.67; 0.96)	0.019	1.44 (1.09; 1.91)	0.009
2012	3.66 (3.14; 4.27)	<0.001	0.58 (0.48; 0.69)	<0.001	0.88 (0.66; 1.15)	0.401
HIV status unknown						
2009	0.82 (0.74; 0.90)	<0.001	1.25 (1.12; 1.40)	<0.001	0.88 (0.68; 1.15)	0.346
2010	0.86 (0.78; 0.95)	0.005	1.12 (0.98; 1.28)	0.099	0.60 (0.47; 0.78)	<0.001
2011	0.96 (0.88; 1.05)	0.413	1.01 (0.90; 1.14)	0.840	0.64 (0.51; 0.81)	<0.001
2012	0.90 (0.83; 0.98)	0.014	0.70 (0.62; 0.79)	<0.001	0.41 (0.33; 0.52)	<0.001
Sex						
Female	1	-	1	-	1	-
Male	0.46 (0.44; 0.48)	<0.001	0.62 (0.59; 0.66)	<0.001	0.85 (0.77; 0.94)	0.002
Age (continuous, per year)	1.09 (1.09; 1.10)	<0.001	1.08 (1.08; 1.09)	<0.001	1.03 (1.01; 1.04)	<0.001
Age^2^	0.99 (0.99; 0.99)	<0.001	0.99 (0.99; 0.99)	<0.001	0.99 (0.99; 0.99)	<0.001
Area of living						
Rural	1	-	1	-	1	-
Peri-urban	0.94 (0.90; 0.98)	0.009	1.18 (1.11; 1.25)	<0.001	1.16 (1.04; 1.28)	0.007
Urban	0.79 (0.72; 0.87)	<0.001	2.06 (1.85; 2.29)	<0.001	1.51 (1.28; 1.78)	<0.001
Outside DSA	0.48 (0.44; 0.53)	<0.001	1.48 (1.31; 1.66)	<0.001	0.78 (0.62; 0.96)	0.019
**Model summary**						
*N*	56818		56300		57524	
AIC	68363		43827		28286	
BIC	68595		44059		28519	
DF	26		26		26	
Log likelihood	-34155		-21887		-14117	
p-value	<0.001		<0.001		<0.001	

PHC = primary health care, AOR = Adjusted Odds Ratio, AIRR = Adjusted Incidence Rate Ratio, *N* = Number of observations, AIC = Akaike Information Criterion, BIC = Bayesian Information Criterion, DF = Degrees of Freedom, DSA = Demographic Surveillance Area

Logistic regressions for public- and private-sector PHC clinics; Poisson regressions for hospitalization rates.

Models with trends in utilization over time by HIV status, ART status, and calendar year, corrected for sex, age, and area of residence are shown. Both uncorrected and corrected trends are given in S1 Table. All regression models contain random individual effects. Pre-2009 trend in ART coverage was as follows: 0.0% in 2004; 1.0% in 2005; 3.8% in 2006; 8.3% in 2007; 14.3% in 2008 [25]. Coverage in the study period was as follows: 27.2% in 2009; 29.4% in 2010; 35.6% in 2011; and 45.5% in 2012 (Table 1).

## Discussion

We have analyzed the changes in healthcare utilization patterns in more than 30,000 adults living in a community in rural South Africa that has been under continuous health and demographic surveillance during a time of rapid ART scale up. The rapid scale-up of a vertical HIV treatment and care program in rural KwaZulu-Natal, South Africa, coincided with significantly increasing rates of public-sector PHC clinic visits in both HIV-infected and -uninfected people over the period 2009–2012. In contrast, hospitalization rates in the population significantly declined by more than one third in HIV-infected people and by about half for HIV-uninfected people over the same period, and private-sector PHC declined significantly as well.

To our knowledge, this is the first study that examined the population-wide effects of expanding HIV treatment programs on health care utilization in the general population. Our results consistent with studies from Rwanda [30], Uganda [31], and Haiti [32], all showing that the introduction of HIV treatment and care programs in public-sector PHC coincided with an increase in the volume of non-HIV service utilization at these clinics. Changes in the volume of non-HIV services at HIV clinics could also indicate a shift in utilization from clinics not offering ART to clinics offering ART, without any change in the population level of health care utilization or in the distribution of utilization across the levels of health care. The unique combination of a population based health and demographic cohort data linked to patient data from an ART program allowed us to quantify for the first time population-level health care utilization trends among HIV-infected and -uninfected people during a period of rapid ART scale-up.

There are several possible explanations for the observed trends in health care utilization in our cohort. Firstly, the observed declines in hospitalization in HIV-infected people can be explained by the effects of ART in improving patients’ health. As patients are successfully maintained on ART, their CD4 cell counts and overall health improves, reducing the need for hospital based care [19,33]. The strong decline in hospitalization rates of HIV-uninfected people, coinciding with an increase in health care utilization at public-sector PHC clinics, suggests spill-over effects of the ART treatment program to the uninfected population. Similar to the experiences of ‘therapeutic citizenship’ within ART treatment programs—whereby HIV-infected people on ART act as facilitators of other HIV-infected people to access treatment [20]–health improvements and positive experiences of HIV-infected people accessing ART [34] may have facilitated HIV-uninfected people to seek routine care at public-sector PHC clinics rather than at the hospital or at private-sector PHC clinics. In turn, this ‘extended therapeutic citizenship’ and increased public-sector PHC clinic utilization could have reduced hospitalization rates in this population even further through effective early identification and prevention of conditions at the primary care level.

The effects of such ‘extended therapeutic citizenship’ can also explain the shift from private-sector to public-sector primary care for both HIV-infected and -uninfected people. Positive experiences of HIV-infected people utilizing HIV treatment services is likely to result in improved perception of the quality of care at public-sector PHC clinics among both HIV-infected and -uninfected people. As many people—especially in poorer parts in rural South Africa—are still uninsured [17], the knowledge of good quality health care at public-sector PHC clinics passed on by HIV-infected people accessing ART may have reduced the need for the uninfected population to access the expensive private-sector system. Finally, ART is successfully keeping HIV-infected people out of the private-sector due to generally reduced need for health care utilization.

While our findings are consistent with causal effects of the ART scale-up on primary care clinic visits and hospitalization rates, our analyses cannot establish causality. Our causal interpretation is based on the development of health care utilization over time and by HIV and ART status. We cannot rule out that these developments can be explained by factors other than ART scale-up that changed over time in this community in rural KwaZulu-Natal. For instance, national health systems reforms may have changed the availability and quality of primary health care, causing our findings. One major national health systems reform did take place during our observation period, the National Health Insurance reform. However, this reform was only launched towards the end of our observation period (late 2011) [35], and it is therefore unlikely that it can fully explain our findings. Another possible explanation could be the roll-out of other health care programs next to the ART program, for instance maternal- and child-health programs or programs for chronic diseases. However, coverage of childhood vaccination in the area was already high in the years prior to our study period [36] and has not changed substantially [37]. Similarly, trends in cause-specific mortality for non-communicable diseases (NCDs) in the area have not changed during the period of our study [2].

Our results suggest that the ART scale-up may have improved the effectiveness and efficiency of the public-sector health care system [38] because of a reduction in expensive hospital-based care, and possibly earlier identification and successful treatment of diseases at the primary-care level. In addition, both the decline in hospitalization and the shift from private-sector to public-sector health care utilization indicate a decline in the financial burden of health care for the households in this community [39]. Hospital-based care is expensive for families because the district hospital serving this community is located far further from most of the homesteads than the nearest PHC clinic, requiring substantially larger financial outlays for travel to the hospital for patients and the family members who accompany them. While public-sector PHC is free at the point of care, the vast majority of people in this community does not have health insurance and thus needs to pay out-of-pocket to gain access to private-sector primary care. Finally, our results confirm that effective and efficient management of a chronic condition is feasible in this context, and that HIV care can act as a model for other primary care-based cost effective chronic disease management in the future [40]. Further research effort is required to ensure that this potential is maximized.

The delivery model of ART in this area is through stand-alone HIV clinics, yet all HIV clinics are located in immediate proximity and on the premises of public-sector PHC clinics. This delivery model is likely to have facilitated a continuum from vertical to horizontal care delivery because HIV-infected people access ART services could immediately access other routine health care services at the public-sector PHC clinic adjacent to the ART clinic. Further integration of care at the primary level could enhance these effects.

We found that overall self-reported healthcare utilization among men was significantly lower compared to utilization among women for public-sector PHC, private-sector PHC, and hospital-based care (Table 2). Recently, Cornell et al. found that, although HIV-infected men had higher mortality on ART than women, these differences were only partly explained by gender differences in ART initiation, retention, and responses to ART [41]. The authors concluded that these observed gender differences in mortality may best be explained by background differences in mortality unrelated to HIV. As many other studies [42], our study suggests that women utilize health care far more often than men. In as far as this general finding is not explained by differential health care need, one reason for the male-female mortality differential may be the gender differential in health care utilization. In as far as the ART scale-up has enhanced the gender differential in health care utilization, it could also partially explain the widening life expectancy gap between women and men that has been observed following public-sector provision of ART in our study community [43].

Although the ART scale-up has increased access to other health care services over the past years, many barriers for access to care still exist [44,45]. Further improvement of the public-sector PHC system in South Africa will be needed in order to cope with the increasing load of patients in chronic care. As people are successfully maintained on ART, the number of HIV-infected people in care is expected to further increase over the coming years, especially when treatment guidelines will change towards earlier initiation [46,47]. As well as facing one of the world's most severe HIV epidemics, South Africa also faces a high and increasing burden of non-communicable diseases [48–50], which is expected to further increase as the HIV epidemic ages due to ART [51,52]. This changing population-level need for care will require integration of HIV and chronic care for non-communicable diseases (NCD) [53], potentially through alternative, community based approaches of integrated chronic care [54].

Our study has some important limitations. First, we used self-reported health care utilization, which may be inaccurate. However, the general health systems literature shows that health care utilization is usually accurately reported and that recall is particularly good for hospitalization [55]. In addition, there could have been selection bias because health care utilization is only elicited in people who are alive. Given that mortality in this population has dramatically declined due to the ART scale up [2], we would expect that the effect of this potential mortality bias would have amplified the time trends we describe here and that controlling for it would have increased the strength of our main conclusions.

In conclusion, our results suggest that the ART scale-up in a high HIV prevalence community in rural South Africa has shifted health care utilization from hospitals and private-sector primary care clinics to public-sector primary care clinics. This shift is observed for both HIV-infected and -uninfected populations, extending the hypothesis of ‘therapeutic citizenship’, as HIV-infected patients receiving ART facilitate public-sector primary care access for family and community members, regardless of whether they are infected with HIV. The ART scale-up in this community has likely freed up hospital capacity, strengthened the delivery of primary care, and made the overall delivery of care more efficient. Further integration of care at the primary level could harness these effects and ensure that the public-sector health system has sufficient capacity to deal with future health care needs and that families can successfully sustain members in long-term care.

## Supporting Information

S1 Table Multivariable regressions of health care utilization on HIV status, ART status, duration on ART, and calendar year (nested models). Logistic regressions for public- and private-sector PHC clinics; Poisson regressions for hospitalization rates. The Models 1 show the uncorrected trends in utilization over time by HIV status, ART status, and calendar year; the Models 2 show the same trends corrected for sex, age, and area of residence. All regression models contain random individual effects. Pre-2009 trend in ART coverage was as follows: 0.0% in 2004; 1.0% in 2005; 3.8% in 2006; 8.3% in 2007; 14.3% in 2008 [25].
(DOCX)
