# Supplementary material for: The Effect of Antiretroviral Treatment on Health Care Utilization in Rural South Africa: A Population-Based Cohort Study
Source: PLoS One. 2016 Jul 6;11(7):e0158015. doi: 10.1371/journal.pone.0158015 (PMC4934780; doi:10.1371/journal.pone.0158015)
Supplement: S1 Table — Logistic regressions for public- and private-sector PHC clinics; Poisson regressions for hospitalization rates. The Models 1 show the uncorrected trends in utilization over time by HIV status, ART status, and calendar year; the Models 2 show the same trends corrected for sex, age, and area of residence. All regression models contain random individual effects. Pre-2009 trend in ART coverage was as follows: 0.0% in 2004; 1.0% in 2005; 3.8% in 2006; 8.3% in 2007; 14.3% in 2008 [25]. (DOCX) [file pone.0158015.s001.docx]

**S1 Table. Multivariable regressions of health care utilization on HIV status, ART status, duration on ART, and calendar year**

| **Parameter** | **Public-sector PHC clinic visits** | | | | |  |  | **Private-sector PHC clinic visits** | | | | | |  |  | **Hospitalization rate** | | |  | |  |
| --- | --- | --- | --- | --- | --- | --- | --- | --- | --- | --- | --- | --- | --- | --- | --- | --- | --- | --- | --- | --- | --- |
|  |  | *Model 1* | |  | *Model 2* | |  |  | | *Model 1* | |  | *Model 2* | |  |  | *Model 1* | |  | *Model 2* | |
|  | AOR (95% CI) | | p-value | AOR (95% CI) | | p-value |  | AOR (95% CI) | | | p-value | AOR (95% CI) | | p-value |  | AIRR (95% CI) | | p-value | AIRR (95% CI) | | p-value |
| HIV - |  | |  |  | |  |  |  | | |  |  | |  |  |  | |  |  | |  |
| 2009 | 1 | | - | 1 | | - |  | 1 | | | - | 1 | | - |  | 1 | | - | 1 | | - |
| 2010 | 1.03 (0.97; 1.09) | | 0.318 | 1.06 (0.99; 1.12) | | 0.085 |  | | 0.80 (0.74; 0.87) | | <0.001 | 0.80 (0.74; 0.87) | | <0.001 |  | 0.70 (0.60; 0.83) | | <0.001 | 0.70 (0.59; 0.83) | | <0.001 |
| 2011 | 1.22 (1.16; 1.30) | | <0.001 | 1.28 (1.20; 1.36) | | <0.001 |  | 0.74 (0.68; 0.80) | | | <0.001 | 0.72 (0.66; 0.78) | | <0.001 |  | 0.68 (0.58; 0.80) | | <0.001 | 0.68 (0.58; 0.81) | | <0.001 |
| 2012 | 1.33 (1.15; 1.30) | | <0.001 | 1.24 (1.16; 1.32) | | <0.001 |  | 0.54 (0.49; 0.59) | | | <0.001 | 0.52 (0.47; 0.57) | | <0.001 |  | 0.53 (0.44; 0.65) | | <0.001 | 0.52 (0.43; 0.64) | | <0.001 |
| HIV +, no ART |  | |  |  | |  |  |  | | |  |  | |  |  |  | |  |  | |  |
| 2009 | 1.42 (1.27; 1.57) | | <0.001 | 1.30 (1.16; 1.46) | | <0.001 |  | 1.29 (1.13; 1.47) | | | <0.001 | 1.24 (1.08; 1.42) | | 0.002 |  | 1.45 (1.10; 1.92) | | 0.008 | 1.30 (0.99; 1.72) | | 0.062 |
| 2010 | 1.58 (1.42; 1.74) | | <0.001 | 1.49 (1.34; 1.65) | | <0.001 |  | 1.08 (0.95; 1.22) | | | 0.260 | 1.01 (0.88; 1.15) | | 0.918 |  | 1.16 (0.93; 1.44) | | 0.200 | 1.01 (0.81; 1.27) | | 0.918 |
| 2011 | 1.83 (1.66; 2.03) | | <0.001 | 1.71 (1.53; 1.91) | | <0.001 |  | 0.97 (0.84; 1.11) | | | 0.616 | 0.90 (0.78; 1.03) | | 0.121 |  | 0.97 (0.78; 1.20) | | 0.769 | 0.85 (0.69; 1.06) | | 0.152 |
| 2012 | 1.87 (1.68; 2.09) | | <0.001 | 1.69 (1.50; 1.90) | | <0.001 |  | 0.72 (0.61; 0.84) | | | <0.001 | 0.66 (0.56; 0.78) | | <0.001 |  | 0.88 (0.67; 1.14) | | 0.327 | 0.76 (0.58; 0.99) | | 0.041 |
| On ART ≤6m |  | |  |  | |  |  |  | | |  |  | |  |  |  | |  |  | |  |
| 2009 | 5.95 (3.57; 9.90) | | <0.001 | 4.96 (2.94; 8.37) | | <0.001 |  | 2.48 (1.63; 3.76) | | | <0.001 | 2.15 (1.41; 3.30) | | <0.001 |  | 2.08 (1.19; 3.64) | | 0.010 | 1.80 (1.35; 4.85) | | 0.041 |
| 2010 | 7.70 (4.53; 13.08) | | <0.001 | 6.55 (3.80; 11.30) | | <0.001 |  | 1.74 (1.14; 2.66) | | | 0.010 | 1.48 (0.94; 2.32) | | 0.092 |  | 3.05 (1.62; 5.34) | | 0.001 | 2.56 (1.35; 4.85) | | 0.004 |
| 2011 | 13.07 (6.82; 25.03) | | <0.001 | 11.53 (5.97; 22.27) | | <0.001 |  | 2.10 (1.40; 3.16) | | | <0.001 | 1.85 (1.22; 2.82) | | 0.004 |  | 2.59 (1.64; 4.10) | | <0.001 | 2.18 (1.37; 3.46) | | 0.001 |
| 2012 | 6.41 (3.96; 10.37) | | <0.001 | 5.30 (3.24; 8.66) | | <0.001 |  | 1.18 (0.75; 1.86) | | | 0.463 | 0.93 (0.58; 1.49) | | 0.766 |  | 2.20 (1.19; 3.64) | | 0.002 | 1.78 (1.07; 2.95) | | 0.025 |
| On ART > 6m |  | |  |  | |  |  |  | | |  |  | |  |  |  | |  |  | |  |
| 2009 | 5.27 (4.21; 6.59) | | <0.001 | 3.86 (3.07; 4.87) | | <0.001 |  | 1.64 (1.33; 2.02) | | | <0.001 | 1.31( 1.06; 1.62) | | 0.013 |  | 3.15 (2.15; 4.61) | | <0.001 | 2.67 (1.80; 3.94) | | <0.001 |
| 2010 | 6.36 (5.20; 7.77) | | <0.001 | 4.81 (3.91; 5.92) | | <0.001 |  | 1.16 (0.95; 1.41) | | | 0.135 | 0.89 (0.73; 1.09) | | 0.257 |  | 1.52 (1.13; 2.04) | | 0.005 | 1.27 (0.94; 1.71) | | 0.120 |
| 2011 | 9.63 (7.84; 11.81) | | <0.001 | 7.26 (5.90; 8.94) | | <0.001 |  | 1.03 (0.86; 1.23) | | | 0.723 | 0.80 (0.67; 0.96) | | 0.019 |  | 1.70 (1.30; 2.24) | | <0.001 | 1.44 (1.09; 1.91) | | 0.009 |
| 2012 | 4.99 (4.31; 5.79) | | <0.001 | 3.66 (3.14; 4.27) | | <0.001 |  | 0.76 (0.64; 0.91) | | | 0.003 | 0.58 (0.48; 0.69) | | <0.001 |  | 1.06 (0.80; 1.41) | | 0.688 | 0.88 (0.66; 1.15) | | 0.401 |
| HIV status unknown |  | |  |  | |  |  |  | | |  |  | |  |  |  | |  |  | |  |
| 2009 | 0.78 (0.72; 0.85) | | <0.001 | 0.82 (0.74; 0.90) | | <0.001 |  | 1.18 (1.05; 1.31) | | | 0.004 | 1.25 (1.12; 1.40) | | <0.001 |  | 0.90 (0.69; 1.17) | | 0.418 | 0.88 (0.68; 1.15) | | 0.346 |
| 2010 | 0.69 (0.63; 0.76) | | <0.001 | 0.86 (0.78; 0.95) | | 0.005 |  | 0.91 (0.80; 1.04) | | | 0.158 | 1.12 (0.98; 1.28) | | 0.099 |  | 0.61 (0.47; 0.79) | | <0.001 | 0.60 (0.47; 0.78) | | <0.001 |
| 2011 | 0.78 (0.71; 0.85) | | <0.001 | 0.96 (0.88; 1.05) | | 0.413 |  | 0.86 (0.77; 0.97) | | | 0.013 | 1.01 (0.90; 1.14) | | 0.840 |  | 0.66 (0.52; 0.83) | | <0.001 | 0.64 (0.51; 0.81) | | <0.001 |
| 2012 | 0.73 (0.67; 0.78) | | <0.001 | 0.90 (0.83; 0.98) | | 0.014 |  | 0.59 (0.53; 0.66) | | | 0.004 | 0.70 (0.62; 0.79) | | <0.001 |  | 0.43 (0.34; 0.53) | | <0.001 | 0.41 (0.33; 0.52) | | <0.001 |
|  |  | |  |  | |  |  |  | | |  |  | |  |  |  | |  |  | |  |
| Sex |  | |  |  | |  |  |  | | |  |  | |  |  |  | |  |  | |  |
| Female | - | | - | 1 | | - |  | - | | | - | 1 | | - |  | - | | - | 1 | | - |
| Male | - | | - | 0.46 (0.44; 0.48) | | <0.001 |  | - | | | - | 0.62 (0.59; 0.66) | | <0.001 |  | - | | - | 0.85 (0.77; 0.94) | | 0.002 |
|  |  | |  |  | |  |  |  | | |  |  | |  |  |  | |  |  | |  |
| Age  (continuous, per year) | - | | - | 1.09 (1.09; 1.10) | | <0.001 |  | - | | | - | 1.08 (1.08; 1.09) | | <0.001 |  | - | | - | 1.03 (1.01; 1.04) | | <0.001 |
| Age^2^ | - | | - | 0.99 (0.99; 0.99) | | <0.001 |  | - | | | - | 0.99 (0.99; 0.99) | | <0.001 |  | - | | - | 0.99 (0.99; 0.99) | | <0.001 |
|  |  | |  |  | |  |  |  | | |  |  | |  |  |  | |  |  | |  |
| Area of living |  | |  |  | |  |  |  | | |  |  | |  |  |  | |  |  | |  |
| Rural | - | | - | 1 | | - |  | - | | | - | 1 | | - |  | - | | - | 1 | | - |
| Peri-urban | - | | - | 0.94 (0.90; 0.98) | | 0.009 |  | - | | | - | 1.18 (1.11; 1.25) | | <0.001 |  | - | | - | 1.16 (1.04; 1.28) | | 0.007 |
| Urban | - | | - | 0.79 (0.72; 0.87) | | <0.001 |  | - | | | - | 2.06 (1.85; 2.29) | | <0.001 |  | - | | - | 1.51 (1.28; 1.78) | | <0.001 |
| Outside DSA | - | | - | 0.48 (0.44; 0.53) | | <0.001 |  | - | | | - | 1.48 (1.31; 1.66) | | <0.001 |  | - | | - | 0.78 (0.62; 0.96) | | 0.019 |
| **Model summary** |  | |  |  | |  |  |  | | |  |  | |  |  |  | |  |  | |  |
| *N* | 56818 | |  | 56818 | |  |  | 56300 | | |  | 56300 | |  |  | 57524 | |  | 57524 | |  |
| AIC | 75686 | |  | 68363 | |  |  | 46401 | | |  | 43827 | |  |  | 28394 | |  | 28286 | |  |
| BIC | 75865 | |  | 68595 | |  |  | 46579 | | |  | 44059 | |  |  | 28574 | |  | 28519 | |  |
| DF | 20 | |  | 26 | |  |  | 20 | | |  | 26 | |  |  | 20 | |  | 26 | |  |
| Log likelihood | -37823 | |  | -34155 | |  |  | -23180 | | |  | -21887 | |  |  | -14178 | |  | -14117 | |  |
| p-value | <0.001 | |  | <0.001 | |  |  | <0.001 | | |  | <0.001 | |  |  | <0.001 | |  | <0.001 | |  |

PHC = primary health care, AOR=Adjusted Odds Ratio, AIRR = Adjusted Incidence Rate Ratio, *N* = Number of observations, AIC = Akaike Information Criterion, BIC = Bayesian Information Criterion, DF = Degrees of Freedom, DSA = Demographic Surveillance Area

Logistic regressions for public- and private-sector PHC clinics; Poisson regressions for hospitalization rates. The Models 1 show the uncorrected trends in utilization over time by HIV status, ART status, and calendar year; the Models 2 show the same trends corrected for sex, age, and area of residence. All regression models contain random individual effects. Pre-2009 trend in ART coverage was as follows: 0.0% in 2004; 1.0% in 2005; 3.8% in 2006; 8.3% in 2007; 14.3% in 2008 [25].
